# Supplementary material for: An Essential Role for Alzheimer’s-Linked Amyloid Beta Oligomers in Neurodevelopment: Transient Expression of Multiple Proteoforms during Retina Histogenesis
Source: Int J Mol Sci. 2022 Feb 17;23(4):2208. doi: 10.3390/ijms23042208 (PMC8875314; doi:10.3390/ijms23042208)
Supplement: Supplementary file 1 [file ijms-23-02208-s001.zip › ijms-1596856-Supplemental Material.pdf]

Supplementary Materials

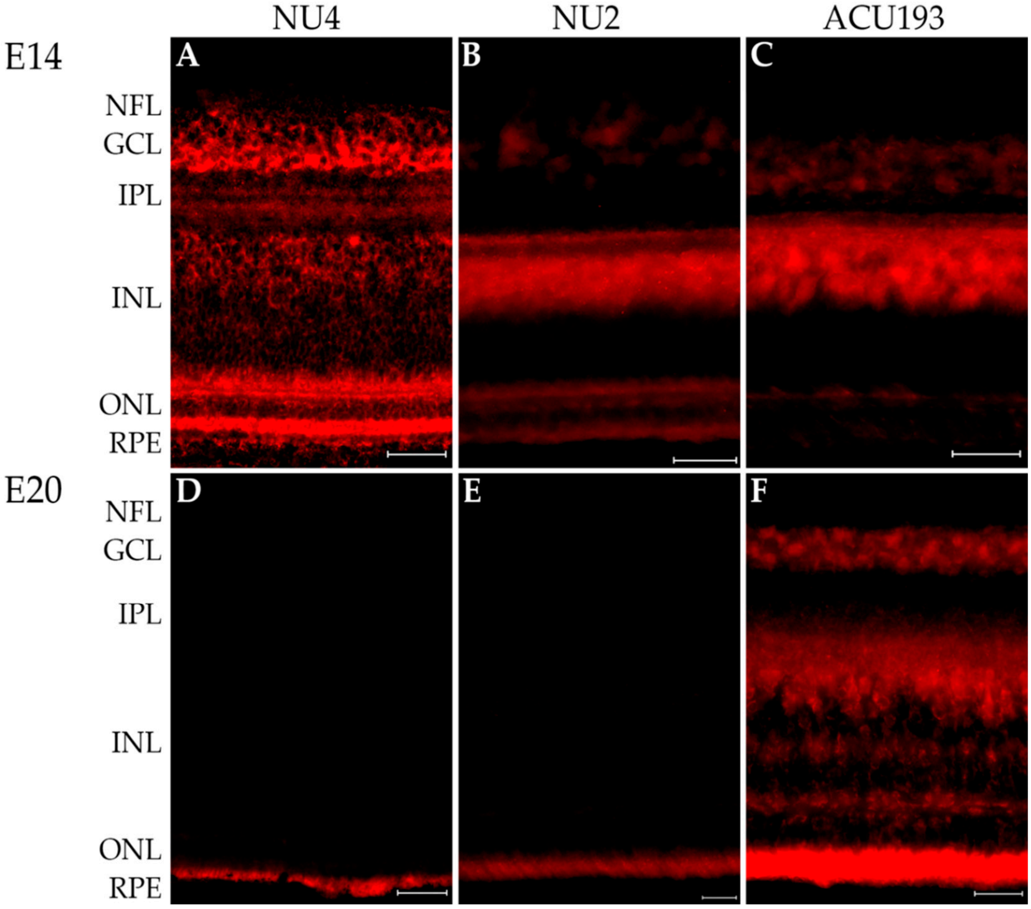

Figure S1. AβO proteoforms targeted by NU4, NU2, and ACU193 show three distinct patterns of spatiotemporal expression. For direct comparison, images shown in Figures 7-9 have been placed side-by-side, showing AβO proteoforms detected by NU4, NU2, and ACU193 at ages E14 and E20 (discussed in text). Scale bar = 25 µm.

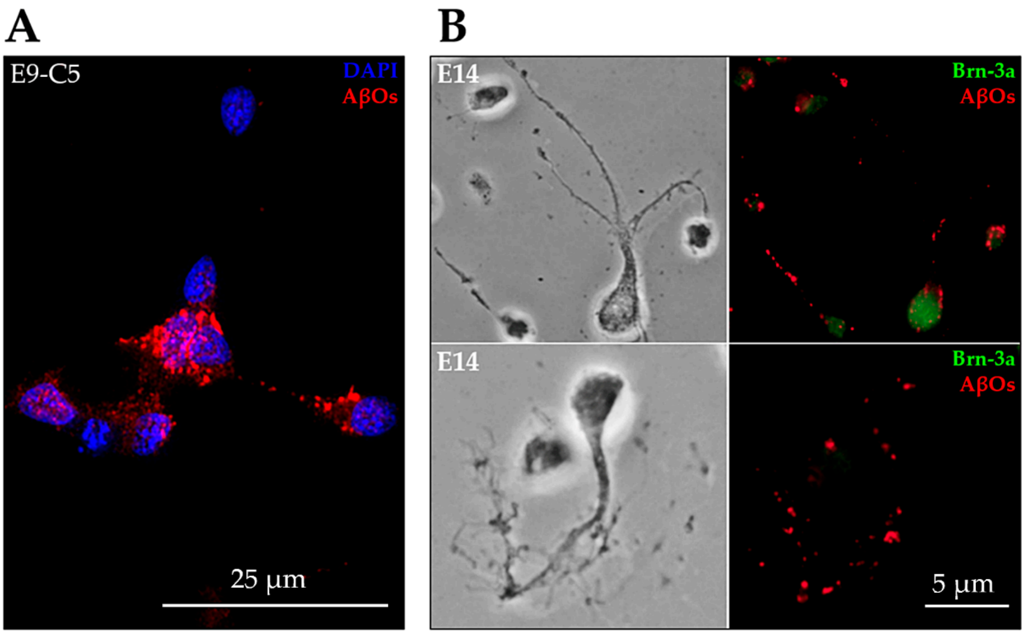

Figure S2. AβOs show punctate cell-selective distribution in soma and neurites of cultured and papain-isolated retina neurons. Isolated retina neurons maintained in cell culture or that were acutely isolated were examined for the presence of AβOs using NU4. (a) AβOs were evident in neurons cultured for 5 days, distributing to puncta found in the soma as well as neurites. Not all cells showed prominent AβOs. Scale bar is 25μm. (b) AβOs in puncta were evident in cells acutely isolated from papain-treated retina, appearing as puncta in Brn-3a-labeled ganglion cells (top) or in neurons unlabeled by Brn-3a (bottom). Scale bar is 5μm.

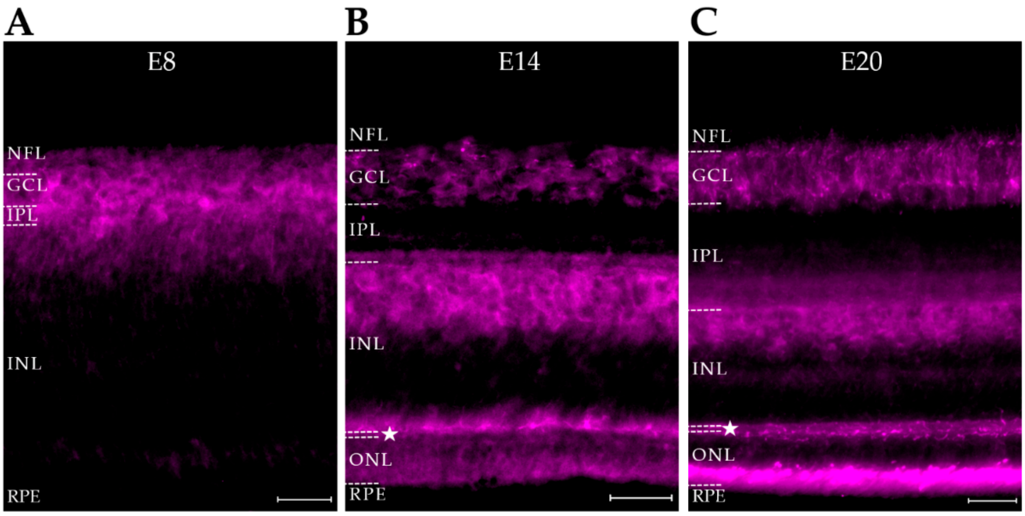

Figure S3. APP expression begins in the inner retina, spreads outward, and is not downregulated at E20. Retinas from embryonic days E8, E14, and E20 were stained for APP using 22C11 (magenta). (a) E8 immunoreactivity is present in the NFL, GCL, nascent IPL, and faintly expressed in the inner INL. (b) E14 APP is dispersed through all layers of the retina, with immunoreactivity distributed to the GCL, the interface between the IPL and INL, flanking the OPL, and in the ONL. (c) E20 immunoreactivity remains high and in the same layers as E14. The OPL is denoted with a star. Scale bar = 25µm. N = 2.

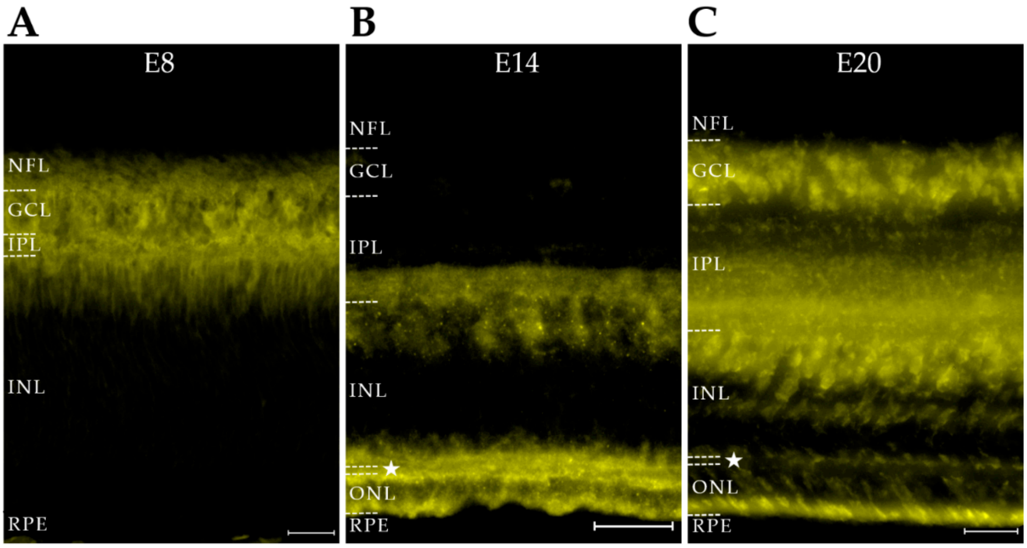

Figure S4. BACE-1 expression is selective and continues through E20. Retinas from embryonic days E8, E14, and E20 were stained for BACE-1 using the rabbit monoclonal antibody D10E5 (yellow). (a) E8 immunoreactivity is present in the NFL, GCL, nascent IPL, and faintly expressed in the inner INL. (b) E14 immunoreactivity is localized to the interface between the IPL and INL, as well as flanking the OPL and the outer ONL. The outer IPL shows specific sub-banding. (c) E20 immunoreactivity is present in a cell-selective manner through all layers of the retina. The OPL is denoted with a star. Scale bar = 25µm. N = 2. BACE-1 expression appears first in inner retina, shifts by E14 to the middle and outer retina, and then reappears in the inner retina by E20.
